# Correlations between geomagnetic field and global occurrence of cardiovascular diseases: evidence from 204 territories in different latitude

**DOI:** 10.1186/s12889-023-16698-1

**Published:** 2023-09-11

**Authors:** Zheng Chai, Yan Wang, Yi-Ming Li, Zhen-Gang Zhao, Mao Chen

**Affiliations:** Laboratory of Heart Valve Disease and Department of Cardiology, West China Hospital, Sichuan University, No. 37 GuoXue Alley, Chengdu, Sichuan 610041 P.R. China

**Keywords:** Geomagnetic field intensity, Cardiovascular disease, Geomagnetic field direction, Geomagnetic disturbance, Cardiovascular risk factors

## Abstract

**Background:**

The correlation between stable geomagnetic fields and unstable geomagnetic activities with mortality, incidence, and prevalence of cardiovascular diseases (CVDs) remains ambiguous.

**Method:**

To investigate the correlations between geomagnetic field (GMF) intensity and geomagnetic disturbance (GMD) and CVDs events in global, long-period scale, global and 204 countries and territories were included on the base of 2019 Global Burden of Disease study (GBD 2019). Data of GMF intensity, GMD frequency, CVDs events, weather and health economic indicators from 1996 to 2019 of included locations were collected. Linear regression and panel data modelling were conducted to identify the correlations between GMF intensity and CVDs events, multi-factor panel data analysis was also generated to adjust the effect of confounding factors.

**Results:**

For the average data during 1996–2019, linear regression model revealed consistent positive correlations between total GMF (tGMF) intensity and mortality of total CVDs [coef = 0.009, (0.006,0.011 95%CI)], whereas negative correlations were found between horizonal GMF (hGMF) intensity and total CVD mortality [coef = -0.010 (-0.013, -0.007 95%CI)]. When considering the time trend, panel data analysis still demonstrated positive correlation between tGMF and total CVDs mortality [coef = 0.009, (0.008,0.009 95%CI)]. Concurrently, the hGMF negatively correlated with total CVDs mortality [coef = -0.008, (-0.009, -0.007 95%CI)]. When the panel models were adjusted for confounding factors, no reverse of correlation tendency was found between tGMF, hGMF and CVDs events. In high-income territories, positive correlation was found between geomagnetic storm (GMS) frequency and mortality of total CVDs [coef = 14.007,(2.785, 25.229 95%CI)], however, this positive trend faded away gradually with the latitude decreasing from polar to equator.

**Conclusions:**

Stable and long-term horizontal component of GMF may be beneficial to cardiac health. Unstable and short-term GMF called GMD could be a hazard to cardiac health. Our results suggest the importance of regular GMF in maintaining cardio-health state and the adverse impacts of GMD on cardiac health.

## Introduction

**Fig. 1:**
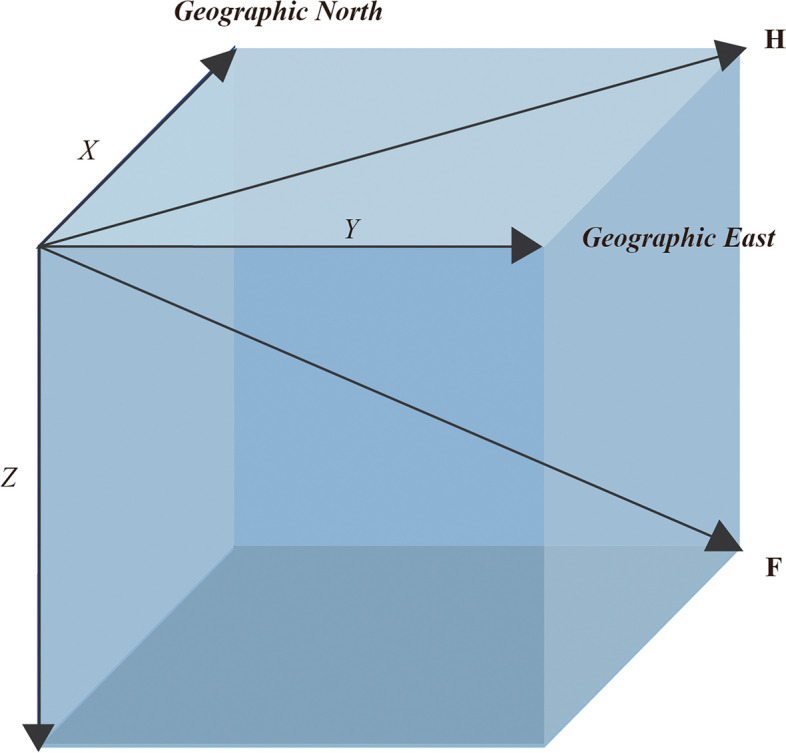
Components of geomagnetic field vector. *Geomagnetic field is described by five parameters, including horizontal intensity (H), vertical intensity (Z), total intensity (F) and the north (X) and east (Y) components of the horizontal intensity. X is the component along the Geographic North direction; Y is the component along the Geographic East direction; Z is the vertical component pointing vertically downwards; H is the magnitude of the horizontal component; F is total geomagnetic field intensity

The Earth's magnetic field, also referred to as the geomagnetic field (GMF), is composed of two components: the Internal Field and the External Field [1]. Internal Field or Main Field is generated by the geomagnetic dynamo process in the Earth’s core, facilitateing the transportation of solar wind particles from the diurnal to nocturnal side of our planet and enabling further spatial redistribution. The Earth’s internal magnetic field is a vector field, and the total GMF intensity (tGMF, F) can be divided into two components: horizontal intensity (hGMF, H) and vertical intensity (vGMF, Z) (Fig. 1). The horizontal components can be described by orthogonal components X (directed towards geographic North), Y (directed towards geographic East). These five components can be interconnected through the following equation:

$$F=\sqrt{{H}^{2}+{Z}^{2}}$$

Where H is given by

$$H=\sqrt{{X}^{2}+{Y}^{2}}$$

**Fig. 2:**
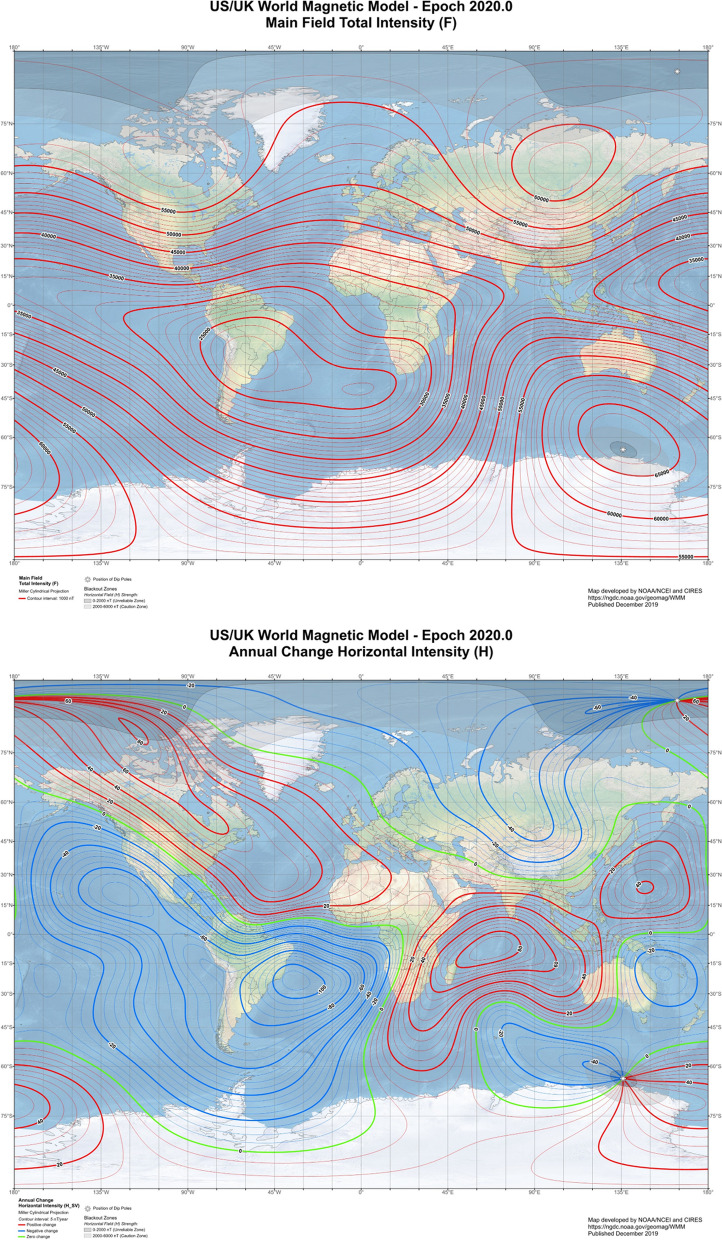
Map of main field total intensity and horizontal intensity from World Magnetic Map (WMM) model for 2020.0. From https://www.ncei.noaa.gov/products/world-magnetic-model

The average total intensity above Earth’s surface ranges from 20,000nT to 65,000nT, depending on latitude and altitude. Considering the vector of the GMF, the horizontal field (H) displays opposite trends compared to the total intensity, being greatest in the equatorial region and weakening towards the Earth's poles [2] (Fig. 2). The external field is highly dynamic and arises from interactions between the solar wind and Earth's internal magnetic field. Some of these interactions can lead to the generation of short-term geomagnetic disturbance (GMD), also referred to as geomagnetic storm (GMS) [3, 4]. These magnetic field disturbances are observable across the Earth's surface and can induce currents, referred to as geomagnetically induced currents (GICs) [5]. Prior research has established that short-term GMDs could have adverse effects on human health, including an increased suicide rate, higher hospitalization rates for acute coronary syndrome, and an elevated risk of stroke occurrences [6–9]. Observations have indicated a strong correlation between solar events and variations in the horizontal geomagnetic component. This correlation suggests that the horizontal geomagnetic field GMF may play a stabilizing role in relation to the charges generated by geomagnetic disturbances GMDs [10–13]. Hence, exploring the connection between environmental physical activities and diverse medical events, as well as understanding how these activities are implicated in specific diseases, can prove valuable for certain aspects of preventive measures. Cardiovascular diseases (CVDs), including conditions like ischemic heart disease (IHD) and stroke, constitute a primary cause of global mortality and a significant contributor to disability [14]. With an electrical conduction system and cardiac muscle cells capable of transmitting electrical stimuli, the cardiovascular system is considered susceptible to the influence of external electromagnetic fields [15–18]. A growing body of research has explored the link between solar and geomagnetic activity (GA) and CVD mortality and morbidity. Regarding short-term exposure to the fluctuating GMF, as mentioned earlier, GMDs have been associated with increased occurrences of total deaths, CVDs, myocardial infarction (MI), cardiac arrhythmia, and stroke events [6, 7, 19–23]. Importantly, certain ground-based investigations have revealed that a hypo-magnetic field (HMF), simulating the low-magnetic environment of outer space, can disrupt circadian rhythms and contribute to the development of mental and physiological disorders [24–26]. Hence, it is plausible to infer that elevated levels of consistent GMF)activity might have beneficial impacts on cardiac health by safeguarding organisms against irregular and transient solar activities.

CVDs exhibited a geographic pattern, characterized by a greater overall incidence of conditions like MI and hypertension in regions situated at higher latitudes [27–29]. While these geographic patterns could be partly attributed to factors such as temperature, ultraviolet exposure, and genetic variations, it remains uncertain whether the intensity of the GMF plays a role as a determinant of CVDs. Furthermore, as previously noted, the horizontal intensity of the GMF is highest at the equatorial region and gradually diminishes towards the Earth's poles.. Consequently, in this original investigation, we aimed to explore the link between stable GA, particularly the horizontal component of the GMF, and CVD risks across 204 countries with varying latitudes. Additionally, we examined the impact of unstable geomagnetic activity, specifically GMDs from solar events, on the occurrence of CVD events.

## Method

### Study design and index definition

As the GMF undergoes periodic fluctuations aligned with the solar cycle (approximately every 12 years), we selected an entire solar magnetic period (1996–2019, encompassing solar cycles 23 and 24) as the timeframe for our investigation [30]. Data of GMF and GMD, Cardiovascular events, meteorological factors, and economic indices from 204 countries or territories globally were gathered from authoritative databases. Both the average values for the entire 24-year period and annual data for each indicator were compiled, enabling separate cross-sectional and panel data analyses.

The countries or territories included in our study consist of 193 sovereign states and 2 quasi-sovereign states, which have been admitted by the United Nations and are members of the World Health Organization (WHO), along with 9 significant economies as defined by the World Bank (https://www.worldbank.org/en/home). Intensity, direction and fluctuation of GMF were all considered in our study, in order to thoroughly investigate the association between GMF and CVD. Apart from total intensity, vertical and horizonal intensity, which were defined as positive in the upward and southward directions respectively, were also taken into account and measured in nanotesla (nT) [31]. Due to the orthogonality relation among the intensities of tGMF, vGMF, and hGMF, our study exclusively analyzed tGMF and hGMF. The frequency of annual strong magnetic disturbances (defined as level G3-G5 by the National Oceanic and Atmospheric Administration, NOAA) was collected for the purpose of fluctuation analysis. This information can be accessed from the NOAA's official website: https://www.swpc.noaa.gov/noaa-scales-explanation [32]. The entire range of CVD, IHD, and stroke were selected as the subjects based on the International Statistical Classification of Diseases and Related Health Problems 10th Revision (ICD-10 2019).

Indicators related to weather and economy were introduced as covariates to mitigate bias. In terms of weather indicators, the maximum and minimum temperatures were measured in degrees Fahrenheit, and the occurrence of specific weather events was quantified using the fractions associated with six distinct conditions (FRSHTT: Fog/Rain/Snow/Hail/Thunder/Typhoon). All countries and territories were categorized based on their gross national income (GNI) per capita into higher income, upper middle income, lower middle income, and low income groups (The World Bank 2020) [33].

### Data source

Data of GMF were obtained based on the 13th generation of the International Geomagnetic Reference Field (IGRF, http://www.geomag.bgs.ac.uk/research/modelling/IGRF.html). For each country or territory, the capital or political center was standardized as the location where the point GMF intensity was recorded, serving as a representation of the general GMF in that region. The specific latitude and longitude information for each location was also obtained from the IGRF model. Data for each individual day spanning from 1996 to 2019 was recorded sequentially and in batches using Node-JavaScript-Runtime, which operated on the server side and facilitated high concurrency. Subsequently, the total and annual GMF intensities were computed for the 204 regions encompassing the 24-year period. Further, the annual frequency of strong GMD was obtained from Space Weather Data Portal (https://lasp.colorado.edu/space-weather-portal/data), employing a filter criteria of G index ≥ 3. Data regarding the FRSHTT ratio, maximum and minimum temperatures were extracted from the National Oceanic and Atmospheric Administration (ftp://ftp.ncdc.noaa.gov/pub/data/gsod/). The daily weather particulars for each region were aggregated into both total and annual data using the same methodology as applied to GMF intensity. The global burden of diseases (GBD) results tool (https://ghdx.healthdata.org/gbd-results-tool) was utilized to access data related to CVDs. This tool serves as a user-friendly platform founded on GBD 2019, offering annual data encompassing a diverse array of measures for all GBD causes, risks, impairments and injuries, across nearly every region globally. The core dataset utilized in this study encompasses 204 countries/territories observed from 1996 to 2019. The selected causes of interest include stroke, IHD, and the total CVDs. Measures of cause include mortality, incidence, and prevalence. For age standardization, the ratio per million people was chosen as the age and metric reference. For each sample, its income level was corresponded according to the GNI. All data underwent independent searching and processing by two researchers, followed by a cross-check to ensure accuracy.

### Statistical analysis

Three main approaches were adopted to ascertain and estimate the effects of GMF on the outcomes. Initially, we present the correlation outcomes yielded by the ordinary least squares (OLS) method, as displayed in Table 1. While it is improbable that our independent variables are influenced by dependent variables, potential unobserved factors that are simultaneously associated with both GMF intensity and CVD events may still introduce disturbances to the results. To address this issue of omitted variables, we utilized both parametric and non-parametric methods to reevaluate our theoretical assumption. The parametric method involved a country fixed-effect model. The merit of this approach lies in its ability to exclude consistent unobserved factors, such as endowments and environmental factors, at the country level through the incorporation of a set of dummy variables. Additionally, mean value imputation, cluster robust standard error and time dummies were utilized to consummate the fixed-effect model. Alternative measurements of GMF (global annual GMF intensity, average GMF intensity of 204 locations) and CVD (total CVDs, IHD and stroke’s mortality, incidence and prevalence) were applied to improve robustness of the estimator in all three approaches mentioned above.

**Table 1:** The correlation results between total intensity (A), horizonal intensity (B) and difference measures of CVD, IHD and stroke in average, single factor panel and multiple-factor panel data analysis

**Variates**	**Average data**			**Single factor panel data**			**Multiple-factor panel data**		
	**Coef (95%CI)**	**R**^**2**^	**P**	**Coef (95%CI)**	**R**^**2**^	**P**	**Coef (95%CI)**	**R**^**2**^	**P**
**A**									
**CVD-M**	0.009 (0.006, 0.011)	0.20	0.000	0.009 (0.008, 0.009）	0.32	0.000	0.005 (0.004, 0.006)	0.43	0.000
**CVD-I**	0.022 (0.017, 0.026)	0.32	0.000	0.019 (0.018, 0.020)	0.51	0.000	0.011 (0.009, 0.012)	0.62	0.000
**CVD-P**	0.160 (0.122, 0.198)	0.25	0.000	0.122 (0.112, 0.131)	0.53	0.000	0.055 (0.047, 0.062)	0.63	0.000
**IHD-M**	0.006 (0.004, 0.007)	0.34	0.000	0.006 (0.005, 0.006)	0.33	0.000	0.004 (0.003, 0.004)	0.44	0.000
**IHD-I**	0.012 (0.009, 0.140)	0.27	0.000	0.011 (0.010, 0.012)	0.41	0.000	0.007 (0.007, 0.008)	0.46	0.000
**IHD-P**	0.086 (0.066, 0.106)	0.26	0.000	0.077 (0.072, 0.082)	0.45	0.000	0.052 (0.047, 0.056)	0.50	0.000
**S-M**	0.002 (0.002, 0.003）	0.12	0.000	0.003 (0.002, 0.003)	0.22	0.000	0.002 (0.001, 0.002)	0.29	0.000
**S-I**	0.003 (0.002, 0.004)	0.17	0.000	0.003 (0027, 0.0033)	0.27	0.000	0.002 (0.0016, 0.0021)	0.35	0.000
**S-P**	0.020 (0.013,0.026)	0.15	0.000	0.015 (0.013, 0.017)	0.36	0.000	0.009 (0.008, 0.011)	0.43	0.000
**B**									
**CVD-M**	-0.010 (-0.013, -0.007)	0.16	0.000	-0.008 (-0.009, -0.007)	0.25	0.000	-0.001 (-0.002, -0.001)	0.39	0.000
**CVD-I**	-0.024 (-0.029, -0.018)	0.26	0.000	-0.018 (-0.020, -0.017)	0.44	0.000	-0.005 (-0.006, -0.003)	0.58	0.000
**CVD-P**	-0.198 (-0.243, -0.152)	0.27	0.000	-0.142 (-0.153, -0.132)	0.53	0.000	-0.055 (-0.064, -0.045)	0.63	0.000
**IHD-M**	-0.006 (-0.007, -0.004)	0.15	0.000	-0.005 (-0.005, -0.004)	0.23	0.000	-0.0004 (-0.0008, 0.0001)	0.39	0.102
**IHD-I**	-0.012 (-0.015, -0.009)	0.20	0.000	-0.009 (-0.010, -0.008)	0.30	0.000	-0.002 (-0.003, -0.001)	0.40	0.000
**IHD-P**	-0.087 (-0.112, -0.061)	0.18	0.000	-0.058 (-0.064, -0.052)	0.36	0.000	-0.010 (-0.016, -0.005)	0.44	0.000
**S-M**	-0.002 (-0.003, -0.001)	0.08	0.000	-0.002 (-0.0023, -0.0019)	0.15	0.000	-0.0001 (-0.0003, 0.0002)	0.26	0.619
**S-I**	-0.003 (-0.004, -0.001)	0.08	0.000	-0.002 (-0.0022, -0.0017)	0.18	0.000	0.0004 (0.0002, 0.0007)	0.31	0.002
**S-P**	-0.018 (-0.026, -0.010)	0.09	0.000	-0.010 (-0.011, -0.009)	0.31	0.000	0.001 (-0.001,0.002)	0.40	0.503

^*^F total intensity, *H* horizonal intensity, *CVD* cardiovascular diseases, *IHD* ischemic heart diseases, *S* stroke, *M* mortality, *I* incidence, *P* prevalence, *Coef* coefficient

To investigate the impact of GMD, linear regression was applied to explore the relationship between global GMS frequency and CVD mortality. Furthermore, our panel analysis exclusively encompassed high-income countries and territories. This choice was made to mitigate potential reporting biases stemming from backward development. Panel data was structured based on location and year. Additionally, we conducted stratified analysis according to latitude (0–15° near-equatorial region, 15–55° mid-latitude region, 55–90° near-polar region) was also conducted.

All the data analysis was processed using STATA (StataSE 16 64-bit), and statistical significance was defined as *P* < 0.05.

## Result

For the cross-sectional study, annual data on GMF intensity and CVD events from 204 included territories, as well as global GMS data, were fully recorded without any gaps occurring during processing. In the panel data analysis, each indicator had 4896 (24*204) data points. Data on CVD events and GMF were complete, while 958 data points were missing for minimum and maximum air temperatures, and 1267 data points were missing for special weather events. All these gaps were filled by using the average values. Additionally, the GNI level was unavailable for 6 territories due to lack of recognized sovereignty.

### Correlation between GMF intensity and CVDs events

**Fig. 3:**
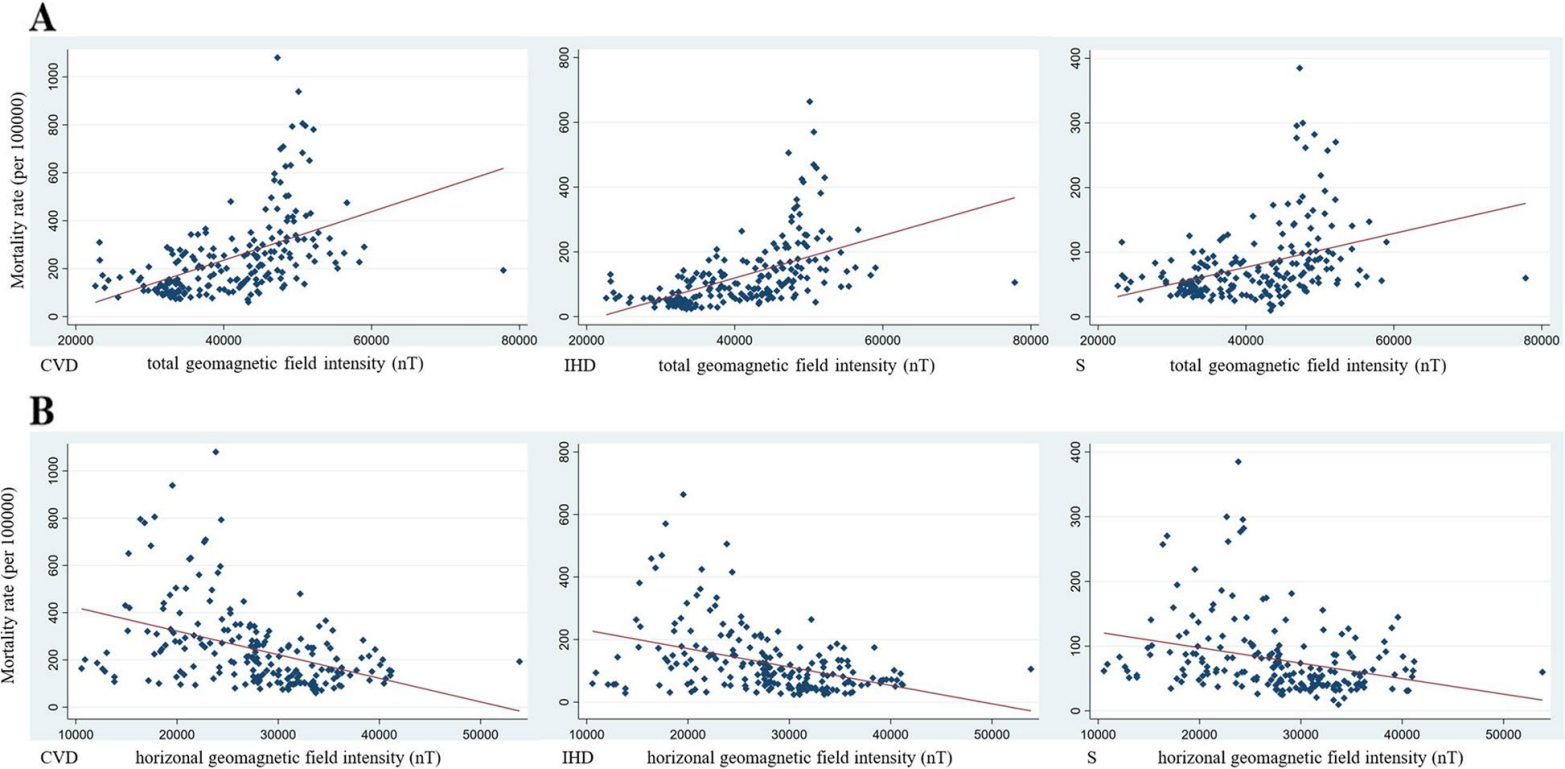
Correlations between mortality rate (per 100000) and geomagnetic field intensity (nT) with total (**A**) and horizonal (**B**) component in CVD, IHD and S.*CVD = cardiovascular diseases, IHD = ischemic heart diseases, S = stroke

In the cross-sectional data analysis using a linear regression model, a significant relationship was observed between GMF intensity and CVD events in terms of mean values. For total CVDs, evident positive correlations were found between tGMF intensity and total CVDs mortality [coef = 0.009, (0.006,0.011 95%CI)], incidence [coef = 0.022, (0.017,0.026 95%CI)] and prevalence [coef = 0.160 (0.122,0.198 95%CI)]. On the contrary, a contrasting pattern emerged with hGMF intensity, showing notable negative correlations with CVD mortality [coef = -0.010 (-0.013, -0.007 95%CI)], incidence [coef = -0.024, (-0.029,-0.018 95%CI)] and prevalence [coef = -0.198, (-0.243,-0.152 95%CI)]. For both IHD and stroke, consistent and conspicuous positive correlations were discovered between tGMF intensity and the rates of mortality, incidence, and prevalence. Conversely, negative correlations were identified between hGMF intensity and the rates of mortality, incidence, and prevalence. The relationships between the mortality rates of CVD, IHD, and stroke, and the intensities of tGMF and hGMF, were effectively illustrated using scatter plots and fitted lines in Fig. 3

The panel data was configured by year and GNI level. In the single-factor panel regression, a positive correlation was observed between tGMF and the mortality [coef = 0.009, (0.008,0.009 95%CI)], incidence [coef = 0.019, (0.018,0.020 95%CI)] and prevalence [coef = 0.122 (0.112,0.131 95%CI)] of total CVDs. On the contrary, hGMF exhibited a negative correlation with total CVDs mortality [coef = -0.008, (-0.009, -0.007 95%CI)], incidence [coef = -0.018, (-0.020, -0.017 95%CI)] and prevalence [coef = -0.142, (-0.153, -0.132 95%CI)]. The correlation trends observed between GMF intensity (tGMF and hGMF) and IHD and stroke events were consistent with the correlation trends seen between GMF intensity and total CVDs. The concurrent association was also evident in multi-factor panel analysis. After accounting for confounding factors, tGMF continued to be identified as a risk factor for total CVDs, IHD and stroke, across mortality, incidence, and prevalence measures. Conversely, hGMF was indicated as a protective factor in all total CVDs events, as well as in the incidence [coef = -0.002, (-0.003, -0.001 95%CI)] and prevalence [coef = -0.010, (-0.016, -0.005 95%CI)] of IHD. Nonetheless, no statistically significant correlation was observed between hGMF and IHD mortality [coef = -0.0004, (-0.0008, 0.0001 95%CI)], stroke mortality [coef = -0.0001, (-0.0003, 0.0002 95%CI)] and prevalence [coef = 0.001, (-0.001,0.002 95%CI)]. No transformation in significance and tendency occurred after the adjustment of time dummies, the comprehensive results of correlations between GMF intensity and CVDs events were illustrated in Table 1.

### Correlation between GMD frequency and CVDs events

Between the years 1996 and 2019, there was no noteworthy linear association detected between global GMD frequency and global CVDs mortality [coef = -0.226 (-0.478,0.170 (95%CI)] However, distinct positive and negative correlations were observed between GMD frequency and stroke mortality [coef = 0.339, (0.185,0.493 95%CI)] and IHD mortality [coef = -0.338, (-0.596, -0.080 95%CI)] respectively.

In our panel analysis, a total of 63 high-income countries or territories were encompassed within our study. Among these entities, 9 were allocated to the near-equatorial region group, 46 were classified under the mid-latitude group, and 8 were segregated into the near-polar region group.. Across all the included territories, GMD exhibited a significant association with elevated mortality rates for CVDs [coef = 14.007,(2.785, 25.229 95%CI)], IHD [coef = 13.688, (6.040, 21.335 95%CI)] and stroke [coef = 8.891, (4.928, 12.855 95%CI)]. For stratification analysis, significant correlations were observed in near-polar territories between GMD frequency and mortality rates of IHD [coef = 39.703, (7.315, 72.092 95%CI)] and stroke [coef = 19.461, (2.314, 36.607 95%CI)]. Comparable findings were obtained for mid-latitude regions, where the presence of GMS was associated with increased mortality rates for total CVDs ([coef = 15.452, (3.131, 27.775 95%CI)], IHD [coef = 14.556, (6.998, 22.114 95%CI)] and stroke [coef = 9.212, (4.853, 13.572 95%CI)]. However, with decreasing latitude and the concurrent decline in GMD frequency alongside the gradual increase in horizontal GMF, the trend of positive correlation between GMD and CVDs events diminished in near-equatorial regions. No significant relationship was observed between GMD frequency and mortality for any type of CVD. This suggests that GMD and horizontal GMF might respectively function as a risk factor and a protective factor for CVD events from an alternative perspective (Table 2).

**Table 2:** Correlations between geomagnetic storm frequency in developed territories with different latitude and difference measures of CVD, IHD and stroke

**Variates**	**Total areas**			**Near-polar region**			**Middle latitude**			**Near-equatorial region**		
	**Coef (95%CI)**	**R**^**2**^	**P**	**Coef (95%CI)**	**R**^**2**^	**P**	**Coef (95%CI)**	**R**^**2**^	**P**	**Coef (95%CI)**	**R**^**2**^	**P**
**CVD-M**	14.007 (2.785, 25.229)	0.20	0.015	41.645 (-2.721, 86.011)	0.52	0.062	15.452 (3.131, 27.775)	0.25	0.015	-17.951 (-50.268, 14.365)	0.18	0.236
**IHD-M**	13.688 (6.040, 21.335)	0.24	0.001	39.703 (7.315, 72.092)	0.59	0.023	14.556 (6.998, 22.114)	0.32	0.000	-13.877 (-36.585, 8.831)	0.18	0.196
**S-M**	8.891 (4.928,12.855)	0.30	0.000	19.461 (2.314, 36.607)	0.53	0.031	9.212 (4.853, 13.572)	0.36	0.000	-2.144 (-11.429, 7.140)	0.15	0.609

^*^GMS-F geomagnetic storm frequency, *CVD* cardiovascular diseases, *IHD* ischemic heart diseases, *S* stroke, *M* mortality, *I* incidence, *p* prevalence

## Discussion

In this international long-term analysis, we studied how tGMF, hGMF, and GMD) frequency affect CVDs. By including countries at different latitudes, we found evidence supporting hGMF's protective role against CVDs. As latitude decreases, GMD risk also reduces. Notably, our study is the first to explore the impact of various Geomagnetic Field components, including vector facets (horizontal and vertical) and contribution facets (internal and external), on CVD events.

Overall, our study demonstrated that both stable and unstable GMF are correlated to CVDs events. As for total intensity of stable GMF, our cross-sectional data showed clear positive correlation between tGMF and CVDs events, and this association remained similar and statistically significant in panel data analysis, which took GNI level and temporal components into consideration. To our knowledge, this study is the first to evaluate the impact of the Earth’s main field intensity on CVD. We used International Geomagnetic Reference Field (IGFR) Calculator to get large-scale, time-varying portion of Earth’s internal magnetic field from 1996 to 2019. Although our results about total GMF are similar to previous epidemiological studies which showed the positive relationship between total GA and CVDs, the meaning of geomagnetic indexes in those studies is totally different from us [19, 20, 34, 35]. In those studies, they used Kp or Ap index, which represents the disturbance of Earth’s magnetic field whereas our study was based on general internal magnetic field in different areas [19–21, 34, 36–39]. GMF protects Earth from solar wind and cosmic rays, stabilizing the ionosphere where Earth’s atmosphere meets the space [9]. However, in our study, total GMF positively related to CVDs events whereas there is an inverse correlation between horizontal GMF (hGMF) and CVDs. It can be due to the deflection of charged particles influenced by different component of GMF [40]. The horizontal GMF produces vertical electric field and exerts a vertical force on atmospheric charged particles, which impedes further separation of the charges. On the contrary, the vertical GMF produces a horizontal electric field and causes charges separated. Our findings about horizontal GMF are consistent with previous study showing the negative relationship between horizontal GMF and multiple sclerosis prevalence [41]. In the present study, we also evaluated the impact of irregular natural GMD. Defined as G-scale ≥ 3 (based on Kp index), GMD showed positive correlations with CVDs in all latitude regions, which are consistent with previous epidemiological studies. Those studies have demonstrated that intense geomagnetic activities (GMA) or GMD increases all-cause, CVD and MI deaths in specific regions, such as Mexico, Russia, Israel and U.S. [20, 34–36, 42]. It is noteworthy that, we included 63 developed countries at multiple latitudes and found those positive correlations diminished as latitude drops and no significant association was observed between GMD frequency and any type of CVDs in subequatorial regions. As shown in Fig. 2, horizontal GMF is greatest around equators and weakest towards poles while total and vertical GMF display opposite arrangement. It is consistent with our main findings that horizontal GMF negatively correlated with CVDs events and supports the idea that horizontal GMF may decrease the risk of adverse CVDs events in intense GMD or GMS.

**Fig. 4:**
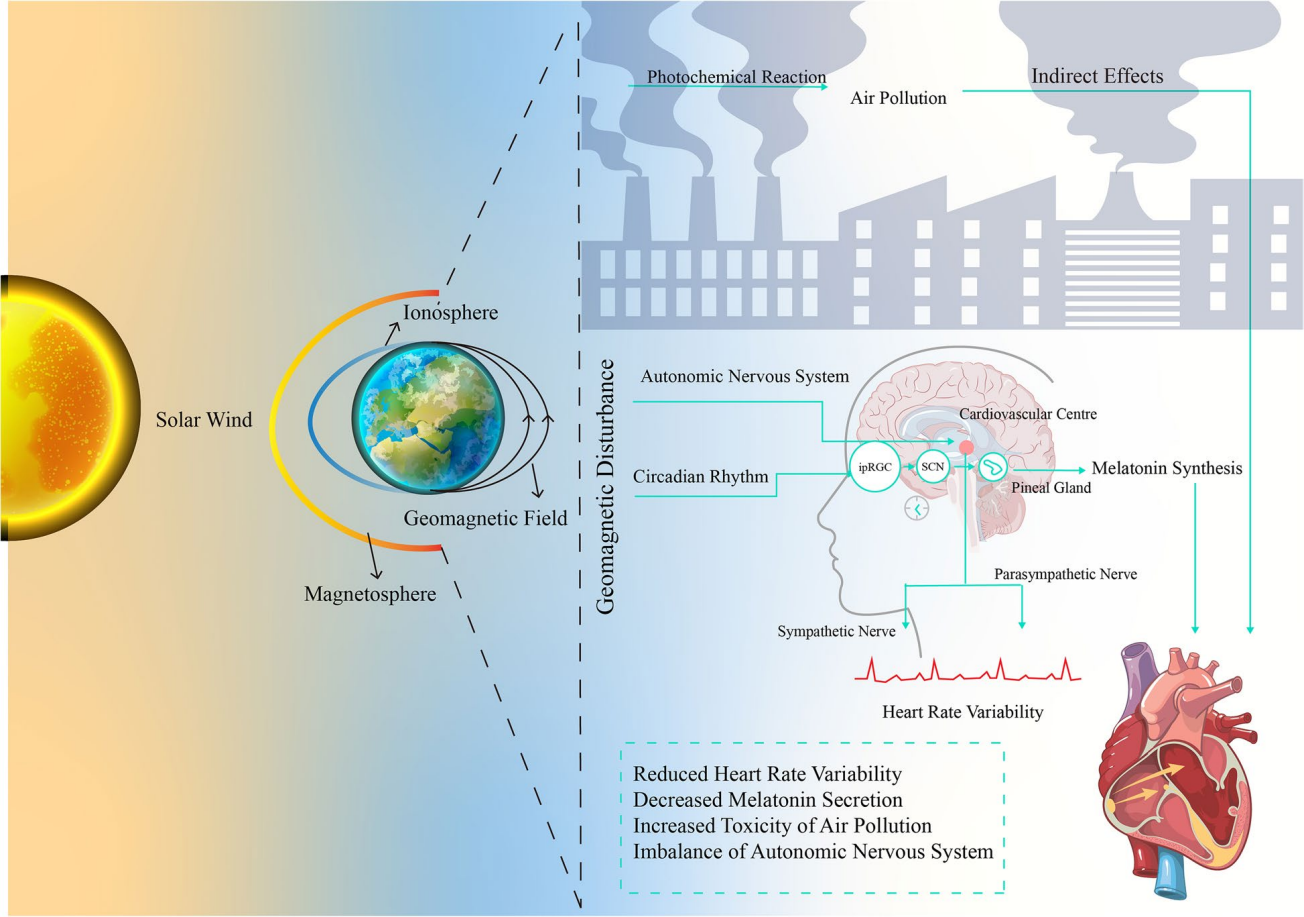
The mechanistic basis of Internal Geomagnetic field and External Geomagnetic disturbance (GMD). *Geomagnetic field protects Earth from ionizing radiation coming from Solar Wind which causes disturbance of electric and magnetic field. Short-term geomagnetic disturbance modulates autonomic nervous system (ANS) and subsequently decreases heart rate variability (HRV). GMD overstimulates photo/magnetic-reception system in intrinsically photosensitive retinal ganglion cells (ipRGCs) and impacts circadian rhythm and melatonin secretion in pineal gland. GMD can alter the toxicity of air pollution which indirectly enhance the association between air pollutants and CVDs events

Understanding the mechanisms by which stable GMF and unstable GMD influence the cardiovascular system is necessary for explaining the results in the present study. The GMF protects Earth’s atmosphere against charged particles from the solar wind, wthereby reducing ionization caused by cosmic rays [43]. Moreover, the notable correlation between horizontal or overall GMF and CVDs, while not robust enough to imply a causal relationship, does necessitate the examination of a potential third variable that could independently influence both factors. GMS is a major disturbance of Earth’s magnetosphere caused by energy exchange between the solar wind and Earth's surrounding atmosphere, resulting in changes in currents, plasmas, and fields within the magnetosphere (NOAA 2022). The perturbation of environmental electric and magnetic fields has the potential to influence the autonomic nervous system (ANS) and circadian rhythm. Several limited-scale cohort studies have showcased a connection between short-term geomagnetic disturbances (GMD) and reduced heart rate variability (HRV). This reduction in HRV serves as an indicator of ANS dysregulation, standing as an established marker for heightened cardiovascular risk [44–46]. Recently, a large cohort of elderly men over a 17-year follow-up demonstrated a significant adverse impact of GA and intense GMD on HRV and the association remained after adjusting for air pollution [19]. Reduced HRV could be the one of the biological mechanisms between GMD and CVDs in our study, however, it remains unclear as to how the body detects GMA variation and GMD. The photo/magnetic-reception system, including cryptochrome protein in retina and ferromagnetic receptors in magnetite-contained cell, is proposed as a mediator enabling organisms to sense variations in the environmental GMF related to GMD [19, 24]. Overstimulating this system disrupts the balance between sympathetic and parasympathetic activities in ANS, which further affects HRV. Furthermore, this system mediates light-dependent stimulation of GMD and influences the melatonin secretion in the pineal gland [47, 48]. Functioning as both an antioxidant and a circadian rhythm regulator, melatonin assumes a vital role in CVDs, encompassing conditions such as MI, ischemic strokes, and arrhythmias [49]. Hence, the mechanisms through which GMD heighten the cardiovascular risk of events entail the dysregulation of the ANS and the reduction of melatonin levels (Fig. 4).

Another potential mechanism for interaction between GMD and CVDs is alterations in the toxicity of air pollution. During periods of GMD, charged particles caused by solar wind can create air ionization and produce electric currents that alter the chemistry of ambient aerosols and droplets [50]. While the precise impact of GMD on altering the electrochemical characteristics and toxicity of ambient particles requires further elucidation, numerous investigations have noted that heightened GMA and pronounced GMD could potentially amplify the connection between air pollutants (such as PM2.5, NO2, black carbon (BC), and ultrafine particles (PN)) and cardiovascular events [7, 23, 50, 51]. Under protection of GMF, these reactions take place on a much smaller scale, which is supportive of our results about the beneficial effects of hGMF on CVDs [9].

There are some limitations in our study. Firstly, the utilization of point data for weather and GMF to represent a country or territory's overall data may introduce biases, particularly in countries with large areas or latitude spans. Secondly, despite the high accuracy verification of the IGRF-13 model (Alken, Thebault et al. 2021), deviations between the model and actual GMF strength do exist. Moreover, the G-index, which is utilized as the sole metric for measuring geomagnetic storms in our study, may introduce potential biases in subsequent analyses due to its inherent limitations and lack of precision. Besides, similar to other studies of this type, the null hypothesis is not independent of the data used for testing. Additionally, it may be difficult to account for all confounding factors and missing data can introduce measurement bias in multi-factor panel analysis. Lastly, although we attested the significant correlations between GMF intensity and CVDs events, the data were not sufficient to demonstrate dose–effect relationship, this is also partly due to inherent limitations in ecological research, such as ‘lack of data on the joint distribution of exposure and disease’, ‘inability to control for potential confounding factors’, and ‘approximate exposure levels in the data’. Future prospective studies addressing such limitations would be needed to substantiate the causal relationship between GMF and CVDs events.

## Conclusion

In conclusion, our study revealed a positive correlation between elevated levels of total GMF intensity and CVDs, alongside a negative correlation between the horizontal component of GMF and CVDs. Furthermore, we identified a positive correlation between unstable GMF activity—GMD—and CVD events. This hypothesis-generating investigation underscores the significance of consistent GMF exposure for maintaining cardiovascular well-being, while highlighting the detrimental effects of GMD on cardiac health. Further prospective studies are imperative to establish a causal relationship between GMF/GMD and CVD events.

## Data Availability

Data of GMF were obtained based on the 13th generation of the International Geomagnetic Reference Field (IGRF, http://www.geomag.bgs.ac.uk/research/modelling/IGRF.html). The annual frequency of strong GMD was obtained from Space Weather Data Portal (https://lasp.colorado.edu/space-weather-portal/data). Data regarding the FRSHTT ratio, maximum and minimum temperatures were extracted from the National Oceanic and Atmospheric Administration (ftp://ftp.ncdc.noaa.gov/pub/data/gsod/). The GBD results tool (https://ghdx.healthdata.org/gbd-results-tool) was utilized to access data related to CVDs. All the included territories’ GNI levels were obtained from the World Bank 2020 (https://www.worldbank.org/en/home).

## Abbreviations

ANS: Autonomic nervous system
BC: Black carbon
CVDs: Cardiovascular diseases
FRSHTT: Fog/rain/snow/hail/thunder/typhoon
GA: Geomagnetic activity
GBD: Global burden of disease
GICs: Geomagnetically induced currents
GMD: Geomagnetic disturbance
GMF: Geomagnetic field
tGMF: Total geomagnetic field
hGMF: Horizonal geomagnetic field
vGMF: Vertical geomagnetic field
GMS: Geomagnetic storm
GNI: Gross national income
HMF: Hypo-magnetic field
HRV: Heart rate variability
ICD-10: International statistical classification of diseases and related health problems 10^th^-revision
IGFR: International geomagnetic reference field
IHD: Ischemic heart disease
MI: Myocardial infarction
NOAA: National oceanic and atmospheric administration
OLS: Ordinary least squares
PN: Ultrafine particles
WHO: World health organization
